# Advances in Nondestructive Technologies for External Eggshell Quality Evaluation

**DOI:** 10.3390/s25185796

**Published:** 2025-09-17

**Authors:** Pengpeng Yu, Chaoping Shen, Junhui Cheng, Xifeng Yin, Chao Liu, Ziting Yu

**Affiliations:** 1 School of Agricultural Engineering, Jiangsu University, Zhenjiang 212013, China; austchengjunhui@126.com; 2 Institute of Aviation Engineering, Jiangsu Aviation Technical College, Zhenjiang 212134, China; shenchaoping0521@163.com; 3 Zhenjiang Agricultural Products Quality Inspection and Testing Center, Zhenjiang 212013, China; insnfeng@163.com; 4 Jurong Agricultural Product Quality and Safety Monitoring Center, Zhenjiang 212013, China; lc880724@163.com (C.L.); 18852881839@163.com (Z.Y.)

**Keywords:** eggshell quality, nondestructive testing, crack detection, strength evaluation, thickness measurement

## Abstract

The structural integrity of poultry eggs is essential for food safety, economic value, and hatchability. External eggshell quality—measured by thickness, strength, cracks, color, and cleanliness—is a key criterion for grading and sorting. Traditional assessment methods, although simple, suffer from subjectivity, low efficiency, and destructive nature. In contrast, recent developments in nondestructive testing (NDT) technologies have enabled precise, automated, and real-time evaluation of eggshell characteristics. This review systematically summarizes state-of-the-art NDT techniques including acoustic resonance, ultrasonic imaging, terahertz spectroscopy, machine vision, and electrical property sensing. Deep learning and sensor fusion methods are highlighted for their superior accuracy in microcrack detection (up to 99.4%) and shell strength prediction. We further discuss emerging challenges such as noise interference, signal variability, and scalability for industrial deployment. The integration of explainable AI, multimodal data acquisition, and edge computing is proposed as a future direction to develop intelligent, scalable, and cost-effective eggshell inspection systems. This comprehensive analysis provides a valuable reference for advancing nondestructive quality control in poultry product supply chains.

## 1. Introduction

Eggs are one of the most commonly consumed animal-derived foods worldwide, valued for their rich nutritional content, moderate cost, and versatile applications in food processing and the culinary industry (Table 1). In both the food industry and agricultural production systems, ensuring the safety and quality of eggs is of paramount importance. Among the various quality factors, the external quality of the eggshell not only directly influences the commercial value and consumer acceptance of the eggs but also affects their resistance to damage and the risk of microbial contamination during the distribution process. Therefore, it serves as a critical indicator in the evaluation of egg quality [1,2].

The eggshell serves as a natural barrier that protects internal egg contents from microbial contamination and mechanical damage, while also supporting embryonic development. Its physical properties directly influence egg safety, hatchability, and market value. However, evaluating eggshell quality remains challenging due to the multidimensional nature of its characteristics—such as thickness, strength, microcracks, and porosity—which are difficult to detect visually and vary significantly between eggs. Traditional inspection methods, including manual observation, tapping, and destructive testing, are prone to subjectivity, have low efficiency, and often damage the eggs. These limitations make them unsuitable for modern egg production, which demands accurate, nondestructive, and automated evaluation. Even some semi-automated acoustic techniques risk inducing micro-damage, further reducing their applicability in high-throughput environments [3,4].

To address these challenges, nondestructive testing (NDT) technologies have emerged as a promising solution for achieving efficient, standardized, and automated eggshell quality evaluation [5]. Advances in image processing [6], machine vision [7,8,9], near-infrared spectroscopy [10,11,12], ultrasound testing [13,14], X-ray imaging [15,16], and artificial intelligence [17,18,19] have enabled the application of NDT methods to assess eggshell thickness, detect cracks, evaluate mechanical strength, and analyze surface integrity with improved precision and speed. These new technologies demonstrate immense potential in enhancing detection accuracy, reducing human interference, and improving detection speed [20]. They also provide technical support for quality grading, risk warning, and process optimization in egg production and processing, particularly in meeting the high-throughput demands of modern egg sorting and packaging assembly lines. The nondestructive testing of eggshell external quality is gradually transitioning from laboratory research to industrial applications.

This review focuses on nondestructive testing (NDT) technologies for evaluating the external quality of eggshells and aims to provide a comprehensive overview of current developments in this field. Specifically, the objectives of this review are fourfold:

(1) To summarize the research progress and working principles of mainstream NDT methods for eggshell quality assessment;
(2) To compare the performance, advantages, and application scenarios of different detection techniques;
(3) To identify current challenges related to detection accuracy, data acquisition, environmental variability, and industrial applicability; and
(4) To explore future development directions, including artificial intelligence integration, multimodal sensing, and system-level optimization.

Through this review, we aim to offer researchers and practitioners a systematic reference for advancing nondestructive eggshell inspection technologies.

Furthermore, although the majority of studies focus on table eggs intended for consumption, the evaluation of external eggshell quality is equally essential for incubation eggs, where shell porosity, thickness, and gas exchange capacity influence embryo development and hatching success. This review therefore covers nondestructive testing methods applicable to both table and incubation eggs.

## 2. Evaluation Indicators of Poultry Eggshell Quality

The stability of egg quality and the feasibility of market circulation largely depend on the physical properties of the eggshell. The eggshell not only serves as a natural barrier between the internal nutrients of the egg and the external environment, but its thickness, mechanical strength, color uniformity, and surface cleanliness also form crucial criteria for egg grading, sorting, and sensory evaluation by consumers. Scientifically and accurately measuring these parameters is of significant importance for advancing the standardization of egg grading, automation in processing, and improving hatchability. Currently, the design and selection of nondestructive testing systems must be based on a deep understanding of the core indicators of eggshell quality. The following analysis will focus on key parameters such as thickness, strength, cracks, color, and cleanliness.

However, traditional methods for assessing these indicators typically rely on manual observation, mechanical measurement, or destructive sampling. These approaches suffer from several limitations, including low detection efficiency, high labor intensity, operator subjectivity, and an inability to provide real-time or large-scale evaluations. Furthermore, destructive techniques, while sometimes accurate, render the eggs unusable for further consumption or incubation, making them unsuitable for modern automated production lines. These limitations highlight the urgent need for the development and application of accurate, efficient, and nondestructive technologies for eggshell quality assessment.

### 2.1. Crack Detection

Eggshell cracks are the most common and critical external defects, severely affecting both the safety of the eggs and hatchability. The types of cracks include visible surface cracks, penetrating structural cracks, and latent fine cracks, with the difficulty of detection increasing with the type. Traditional manual visual inspection is inefficient and highly subjective, making it unsuitable for industrial production line requirements [21].

To address this issue, researchers have developed various nondestructive crack detection technologies, including high-resolution machine vision, infrared thermography, acoustic emission, X-ray imaging, and laser interferometric imaging analysis. Among these, machine vision methods, using image edge extraction and texture analysis, can identify obvious crack features [22,23]. Infrared thermography relies on the anomalous thermal conductivity of cracked areas, which forms temperature contrast in thermal images [24]. Acoustic emission techniques monitor abnormal sound signals generated by the eggshell during micro-vibrations or tapping, capturing structural damage caused by cracks [25,26,27]. X-ray and laser interferometry are effective for detecting more concealed microcracks and penetrating structural damage [28,29]. In recent years, the introduction of deep learning algorithms and multimodal data fusion has provided new directions for robust crack detection [30,31,32].

### 2.2. Eggshell Thickness Measurement

Eggshell thickness is a critical parameter influencing the mechanical stability and gas exchange capability of the eggshell. Thin eggshells are more prone to breakage, while excessively thick eggshells may impair normal embryonic development and hatching efficiency. Traditional thickness measurement methods, such as manual measurements with a vernier caliper after breaking the shell, suffer from issues such as sample destruction, low detection efficiency, and high human error, making them unsuitable for automated and high-throughput detection needs.

In recent years, non-contact nondestructive measurement technologies have been widely researched and applied. Among them, ultrasonic thickness measurement is a typical method that works by calculating the eggshell thickness based on the time delay of high-frequency sound waves propagating between the inner and outer walls of the eggshell [33,34]. This method offers high sensitivity and quick response, making it suitable for real-time thickness monitoring in assembly line environments. Laser distance measurement, utilizing principles such as triangulation or Time of Flight (ToF), non-contact scans the eggshell surface [35]. Laser distance measurement, using triangulation or Time-of-Flight (ToF), can be combined with structured-light or interferometric imaging to accurately reconstruct the local thickness distribution of the eggshell, making the combined system ideal for high-precision modeling and defect diagnosis. In addition, both X-ray transmission analysis and capacitive coupling techniques have demonstrated good detection performance in specific studies [36,37].

### 2.3. Eggshell Strength Assessment

Eggshell strength is a key indicator in assessing the egg’s resistance to compression, transportation stability, and adaptability to mechanical processing. It directly determines the integrity of the egg during logistics, handling, and storage. Traditional crushing tests are widely used for eggshell strength assessment, where vertical pressure is applied until the egg breaks, and the maximum breaking force is recorded to evaluate its mechanical limits. Although this method provides accurate results, it is inherently destructive and unsuitable for assessing commercial-grade eggs.

To meet the demand for nondestructive testing, researchers have developed several alternative techniques, including acoustic impact methods and vibration response analysis. The acoustic impact method applies a short-time excitation (e.g., tapping or pneumatic excitation) to the eggshell and analyzes the echo characteristics and resonant frequencies to assess its structural integrity and strength grade. The vibration response method uses an excitation device and sensors to measure the dynamic spectral characteristics of the eggshell, extracting its elastic modes and attenuation factors. These methods allow for the indirect assessment of the eggshell’s compressive strength without damaging the sample and have been preliminarily implemented in several automated grading systems. In the future, the integration of deep learning-based signal pattern recognition will further enhance their robustness in detecting complex samples [38,39,40,41].

### 2.4. Color and Cleanliness Detection

The color and cleanliness of the eggshell are important indicators of its quality and consumer appeal. Consistent color and a clean surface not only enhance the esthetic value of eggs but also influence consumer purchasing decisions. Variations in eggshell color, caused by factors such as breed, diet, and environmental conditions, may affect the perceived freshness or quality of the egg [42]. Additionally, the presence of dirt, feces, or other contaminants on the eggshell surface can lead to microbial contamination, posing a risk to food safety [43]. Therefore, reliable and efficient methods for color and cleanliness detection are essential for maintaining product quality.

Traditional methods for assessing eggshell color and cleanliness often rely on visual inspection or manual grading, which can be subjective, time-consuming, and prone to human error [44]. To address these limitations, recent advancements in nondestructive testing technologies have introduced automated, high-throughput solutions. Modern detection systems commonly utilize image processing techniques and color space transformation methods [45]. After capturing the eggshell image with a high-resolution camera, the system can perform color space analysis (such as RGB to HSV, Lab, etc.), enabling the quantification of color differences and grading. Additionally, by combining image enhancement, grayscale equalization, and threshold segmentation algorithms, these systems can effectively detect surface defects such as stains, cracks, and discolored deposits. In more advanced systems, convolutional neural networks (CNNs) and other deep learning models have been applied for the automatic classification and anomaly detection of eggshell defects, providing enhanced texture representation and discriminative power [46,47,48]. When combined with multispectral imaging or polarization-enhanced techniques [49], the system can significantly improve detection stability and accuracy under complex lighting conditions, thus meeting the practical application needs of high-standard egg sorting.

### 2.5. Other Indicators

In addition to core parameters such as thickness, strength, cracks, color, and cleanliness, other physical characteristics of the eggshell also play a crucial role in determining its overall quality, safety, and market performance. In recent years, with the development of detection technologies and the increasing sophistication of analysis requirements, both academia and industry have begun to pay more attention to the “secondary indicators” of eggshells, such as shape integrity, surface roughness, pore structure, and eggshell mass per unit area [50,51,52].

The geometric shape and integrity of the eggshell are among the key visual attributes used to assess whether the egg meets grading standards [53]. In an ideal state, a typical egg should have an elliptical shape, with its long-to-short axis ratio, shell symmetry, and roundness at the top/bottom serving as key evaluation criteria. Abnormal shapes such as flattened, malformed, or locally collapsed eggs may indicate disruptions in the egg-laying process or developmental anomalies, which not only affect mechanical compression resistance but also hinder packaging adaptation and transportation stability [54]. Modern detection methods can achieve precise geometric analysis through contour extraction, edge fitting, image projection, and morphological parameter calculation, combined with machine vision systems for high-speed online screening [55].

Surface roughness is an important indicator that affects the texture, cleanliness, and antibacterial properties of the eggshell [56]. A rough surface can lead to incomplete disinfection, increased microbial adhesion, and an elevated risk of spoilage. Traditional roughness evaluation methods rely on subjective tactile feedback or microscopic observation. However, in recent years, nondestructive techniques such as 3D reconstruction imaging, laser confocal scanning, and grayscale texture analysis have been introduced to quantify surface microstructures and roughness characteristics [57]. For example, the statistical analysis of grayscale gradients in surface images can be used to construct roughness feature vectors, which, when combined with AI models, can assist in classifying eggshell materials [58].

Pore structure is particularly critical in the hatching process of breeder eggs. The pores in the eggshell are responsible for gas exchange during embryo development, and their number, density, and pore size distribution directly affect the oxygen supply and moisture evaporation rate, which in turn determine the stability of the embryonic growth environment. Currently, the detection of such parameters is primarily carried out in laboratory settings using high-precision imaging devices, such as Scanning Electron Microscopes (SEM) and Micro-Computed Tomography (Micro-CT). Some studies have explored using infrared absorption rates or humidity diffusion rates to infer pore density, aiming to find rapid and nondestructive alternatives [59,60,61,62].

Additionally, some researchers have proposed using eggshell mass per unit area as an alternative indicator that combines both strength and thickness. This measurement relies on high-precision weighing and geometric parameter estimation, making it suitable for automatic acquisition in sorting equipment. By integrating the multidimensional features mentioned above, the future trend of nondestructive testing is gradually shifting from “single-parameter monitoring” to “multidimensional joint evaluation,” supporting the design of more refined and intelligent quality control systems [62].

## 3. Poultry Egg Crack Detection

**Figure 1:**
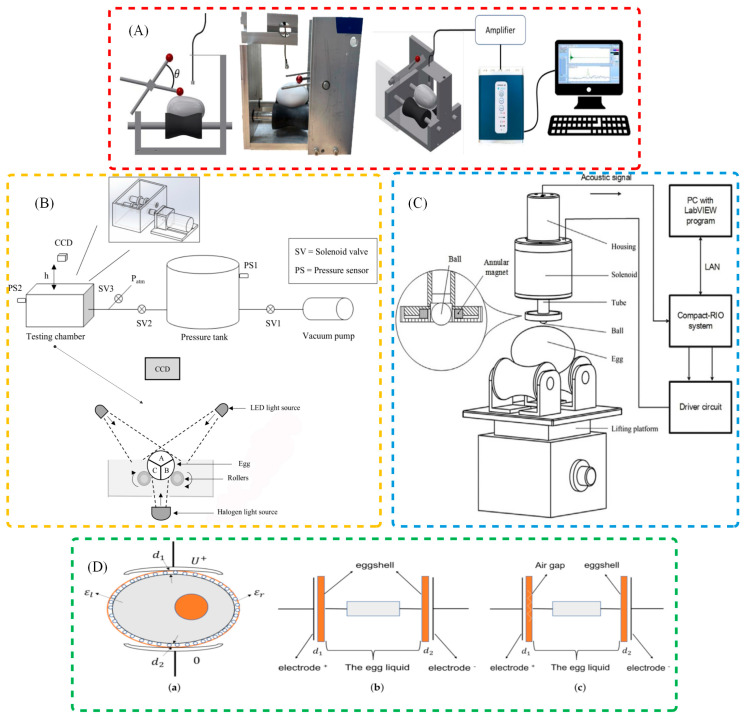
Principles of various techniques for detecting eggshell cracks. (**A**) Schematic of measurement setup: Acoustic detection device for poultry egg cracks, percussion rod action device schematic diagram, and actual device [63]. (**B**) Structure of the vacuum-assisted eggshell crack detection system [64]. (**C**) Schematic diagram and real setup of the acoustic-based eggshell crack detection system [65]. (**D**) Schematic diagrams of the equivalent capacitance model of the egg–electrode system under different crack conditions. (**a**) Capacitor system composed of electrode and egg; (**b**) Equivalent capacitance when electrode is not aligned with the crack; (**c**) Equivalent capacitance when electrode is aligned with the crack [66].

To meet the increasing demands of precision and automation in poultry egg quality assessment, a range of nondestructive technologies have been developed to detect cracks with improved accuracy and efficiency. Building upon traditional image-based techniques, researchers have progressively introduced advanced methods integrating artificial intelligence, acoustic analysis, sensor fusion, and electrical signal processing. These approaches aim to overcome the limitations of conventional inspection systems and offer scalable solutions adaptable to complex production environments. A comparative summary of various eggshell crack detection techniques, along with their key performance metrics, is presented in Table 2. To provide a clearer understanding of the working mechanisms, the principles of various techniques for detecting eggshell cracks are illustrated in Figure 1.

**Table 2:** Comparative summary of eggshell crack detection techniques and performance metrics.

Type	Technique	Parameters	Model	Accuracy	Explanation	Ref.
Image processing (*n* = 5)	Modified pressure imaging system	3 replicates, 360 eggs per replicate, Negative pressure (~200 mmHg, 0.5 s), digital imaging	None	99.60%	Uses negative pressure to expand cracks and detect them through digital imaging.	[67]
	OpenCV-based image processing	CCD camera, R-channel extraction, median filter	None	>90%	Separates color channels and applies filtering and morphological analysis.	[68]
	Canny edge detection + Hough line transform	80 in total, including 45 healthy eggs and 35 cracked eggs, Canny edge detector, Hough line transform	LDA	90.10%	Edge detection and line transformation combined with linear discriminant analysis.	[69]
	Wavelet transform + PCA reduction + SVM classification	48 in total, 24 with artificially created cracks, 24 as controls, Wavelet transform (sym4, 2-layer), PCA reduction	SVM	93.75%	Multi-feature extraction and classification using wavelet transform and PCA.	[70]
	Rotating mechanism + DoG + median filter	50 in total, 30 with artificially created microcracks, 20 intact; 3 images per egg (150 images total), 750 surface images after 5 hold-out validations (450 for training set, 300 for test set), Rotating egg, DoG + median filter	ANN	98%	Uses a rotating mechanism and feature extraction for crack detection.	[64]
Deep learning (*n* = 4)	Multiple CNNs (YOLOv4/v7, Faster R-CNN, SSD)	536 original images, 1280 images after preprocessing and augmentation (1116 for training set, 109 for validation set, 55 for test set), CLAHE preprocessing, YOLOv7	YOLOv7	mAP 0.792	Compares multiple CNN architectures for crack detection.	[71]
	Transfer learning with VGG16/VGG19	569 images in total, including 169 of cracked eggs, 200 of empty eggs, 200 of intact eggs, RGB images, VGG16/VGG19	VGG19	95.10%	Multi-class crack classification using transfer learning.	[72]
	Custom patch-wise CNN	Training set: 216 cracked eggs, 122 intact eggs, 1920 image patches (10,000 after augmentation, 5000 each for cracked and intact); Test set: 65 cracked eggs, 65 intact eggs, 1300 image patches, Grayscale image patches, custom CNN	Custom CNN	95.38%	Custom CNN trained on grayscale image patches.	[73]
	Image stitching + MobileNetV3_egg	800 in total (400 cracked preserved eggs, 400 intact preserved eggs); 1200 images per splicing scheme (400 of each type), Image stitching, MobileNetV3_egg	MobileNetV3_egg	96.30%	Detects cracks in high-throughput settings using image stitching.	[74]
Acoustic detection (*n* = 9)	FPGA-controlled tapping + IGWO optimization	300 in total, 150 with artificially created cracks, 150 intact; 2600 signal samples (1300 each for cracked and intact), Tapping signal, IGWO optimization	IGWO-LightGBM	96.64%	Acoustic signal detection with optimized classification.	[75]
	Rolling acoustic signals on inclined plate	438 in total, including 146 intact eggs, 146 hairline cracked eggs, 146 star cracked eggs, Inclined plate rolling, impulse response	Neural Network	92.3% (Inclined plate), 94.6% (Impulse)	Dual-mode acoustic excitation for crack detection.	[76]
	Solenoid-driven mechanical excitation	Training set: 200 (100 cracked, 100 intact); Test set: 500 (250 brown-shelled, 250 white-shelled, 150 with cracks each), Time/frequency features, F-ratio	Neural Network	99.20%	Acoustic signal detection with feature optimization.	[65]
	Defined bandwidth sound signal acquisition	Calibration group: 60 (30 cracked, 30 intact); Validation group: 66 (34 intact, 32 cracked); 300 data points for calibration group, 330 for validation group (5 taps per egg), Frequencies (1500–10,000 Hz)	Logistic Regression	Training: 89.7%, Prediction: 87.6%	Acoustic signal regression analysis for crack detection.	[63]
	Full-spectrum acoustic spectroscopy	705 measurements total, 693 valid after removing incorrect files; 20 tested weekly for 6 weeks, Spectrum shape, dominant frequencies	Not specified	97.9% (2.1% classification error)	Analyzes broadband frequency features for crack detection.	[77]
	Equatorial excitation acoustic impulse response	Training set: 200 (100 cracked, 100 intact); Test set: 150 (55 cracked, 95 intact); Validation experiment: 240, Frequency-domain features	SVM	98% (up to 98.77%)	Acoustic impulse response with feature fusion.	[78]
	Acoustic resonance with Pearson correlation	1st batch: 25 chicken eggs, 25 duck eggs; 2nd batch: 4 chicken eggs, 4 duck eggs; 3rd batch: 100 chicken eggs, 100 duck eggs, Pearson coefficient, MANOVA	Linear Discriminant Function	95.50%	Mixed-species analysis using acoustic resonance.	[79]
	High-speed mic + CVA classifier	60 intact eggs 59 cracked eggs 10,000 signal samples per egg, Statistical signal features	CVA, ANN, SVM	100% (CVA)	Mechanical tapping and signal classification.	[80]
	Triple-directional vibration sensing	200 total eggs (100 intact + 100 cracked) 120 calibration eggs (60 intact + 60 cracked) 80 prediction eggs (40 intact + 40 cracked), Multiple impacts, DI values	LDA	83.75–93.75%	Vibration signal correlation analysis for crack detection.	[81]
	Force-assisted acoustic sweeping + PCA	180 micro-cracked eggs, 160 intact eggs, Pressure: 5 N, Sweep: 3–7.5 kHz	LS-SVM, BPNN, PNN	LS-SVM: 98.3% (intact), 95% (cracked)	Force-assisted acoustic scanning for crack detection.	[82]
Multi-sensor fusion (*n* = 1)	Fusion of CVS and ARS	300 training eggs (100 intact + 100 cracked) 50 testing eggs (25 intact + 25 cracked) 200 validation eggs (100 intact + 100 cracked), Acoustic parameters + geometric vision metrics	BPANN	CVS: 68%, ARS: 92%, Fusion: 98%	Data-level fusion of computer vision and acoustic response.	[83]
Electrical detection (*n* = 2)	Static and dynamic electrical modeling	770 total eggs (367 intact + 403 cracked) 267 duck eggs (130 intact + 137 cracked), 1500 V DC excitation, feature domains (TF, FF, WF)	Random Forest, SVM, LDA, DT	Random Forest: >99%	Electrical signal feature fusion for microcrack detection.	[66]
	Electric discharge detection	500 total eggs 100 medium 100 large 100 extra-large 100 jumbo 100 processed eggs, 3000 V electric pulse, 15 kHz square waveform	None (physical discharge-based logic)	High visual precision, verified by spark location	Crack detection using high-voltage scanning and electrode array.	[84]

### 3.1. Traditional Vision for Crack Detection

Computer vision technology utilizes high-resolution cameras to capture eggshell images and applies image processing algorithms to analyze cracks [85]. Image processing has long been one of the key approaches in the field of poultry egg crack detection. Backlighting is commonly used to enhance the visibility of cracks on the eggshell, making them more prominent in the captured images and easier for detection systems to identify. Early image processing methods primarily relied on basic image enhancement and threshold segmentation. However, with the advancement of technology, more sophisticated and accurate algorithms have been developed to improve detection precision and reliability. The advantage of this method lies in its non-contact nature and high efficiency, making it particularly suitable for large-scale online detection in industrial production. Common computer vision techniques include image enhancement, edge detection, image segmentation, and feature extraction [86].

For example, Jones et al. [67] developed a modified pressure system that enabled the visualization of microcracks by creating a negative pressure environment, thereby making otherwise imperceptible cracks visible to the naked eye [67]. The successful implementation of this technique significantly enhanced the detection sensitivity for microcracks and has since been widely adopted in advanced production lines. Li et al. [68] proposed a machine vision-based crack detection method that enhances crack visibility through image preprocessing, edge detection, and feature extraction. The extracted features are then classified using a Support Vector Machine (SVM), achieving a detection accuracy of over 90% [68]. Abbaspour-Gilandeh et al. [69] proposed a computer vision-based crack detection method using Hough Transform. The approach begins with image preprocessing and employs the Canny edge detection algorithm to extract edge features from the eggshell image. The Hough Transform is then applied to detect straight lines in the image, under the assumption that cracks exhibit linear characteristics. Finally, Linear Discriminant Analysis (LDA) is used to classify intact and cracked eggs. This method achieved a recognition accuracy of 90.1% in experiments and demonstrated fast detection speed, averaging 0.7 s per egg [69].

With advancements in algorithms, methods such as wavelet transform and Principal Component Analysis (PCA) have been incorporated into crack detection. These techniques, through multi-scale analysis, feature extraction, and dimensionality reduction, have further enhanced detection accuracy and robustness. Xiong et al. [70] proposed a method based on image waveform transformation and multi-feature synthesis, which improved detection performance by extracting spatial frequency domain features of cracks and combining them with PCA [70]. However, in practical applications, image processing methods still face challenges due to the complex and variable surface conditions of eggshells. Stains, scratches, and other interference factors often affect the accuracy of detection results. Particularly when dealing with irregular cracks, fluctuations in image quality can lead to misjudgments [64].

### 3.2. Vision and Deep Learning for Crack Detection

With the development of artificial intelligence technologies, deep learning-based crack detection methods for poultry eggs have been widely adopted. Deep learning models, such as Convolutional Neural Networks (CNNs), can automatically extract high-level features from large volumes of images and perform efficient classification [87]. To overcome the low detection accuracy caused by issues such as uneven lighting, surface stains, and the small size of cracks in traditional image processing methods, many researchers have begun combining deep learning with image processing techniques, utilizing deep learning models such as Convolutional Neural Networks (CNNs) for automatic crack identification. Compared to traditional image processing and acoustic detection methods, deep learning techniques are better suited for handling crack detection tasks in complex backgrounds, particularly excelling in the detection of microcracks [71].

Caballero et al. [71] proposed a crack detection model for duck eggs based on Convolutional Neural Networks (CNNs), comparing various CNN architectures such as YOLOv7, Faster R-CNN, SSD, and RetinaNet. The results showed that YOLOv7 outperformed the others in both accuracy and speed, achieving a mean Average Precision (mAP) of 0.792 [71]. Taspinar et al. [72] trained deep learning models, such as VGG16 and VGG19, on a dataset of 569 eggshell images, successfully automating crack detection. The VGG16 model achieved a classification accuracy of 93.8% [72]. Botta et al. [73] proposed a CNN-based crack detection method that trains the model by extracting various image patches from eggshell images, achieving an accuracy of 96.92%. This method not only effectively identifies more complex crack patterns but also significantly improves detection speed and robustness [73]. Tang et al. [74] proposed a deep learning model based on an improved version of MobileNetV3 for crack detection in preserved eggs. The method enhances model robustness and accuracy by stitching images and performing data preprocessing, combining eggshell images from multiple angles into a complete detection dataset. Experimental results showed that the MobileNetV3_egg model achieved a detection accuracy of 96.3%, with a processing time of only 4.267 s per 300 images, fully meeting the requirements for online detection [74]. In conclusion, deep learning technologies are particularly well-suited for handling large volumes of image data and can effectively identify microcracks in complex backgrounds [88]. With the continuous optimization of deep learning algorithms and the expansion of training datasets, future crack detection systems will be able to handle more complex and larger-scale detection tasks, while improving both their real-time performance and accuracy.

### 3.3. Acoustic for Crack Detection

Acoustic detection technology is a nondestructive method that detects cracks by analyzing the acoustic response of poultry eggs under stress. This technique generates sound waves through slight impacts or vibrations, and then analyzes the propagation characteristics of the sound waves to assess the integrity of the eggshell [89]. Acoustic signals can reveal cracks on the eggshell surface, particularly being more sensitive than image processing methods in detecting microcracks [75]. The characteristics of the acoustic signals are typically closely related to the depth, location, and type of the crack. By extracting features and using pattern recognition, cracked eggs can be effectively distinguished from intact eggs. Unlike image processing, acoustic detection does not rely on external light sources, allowing it to effectively overcome lighting limitations. As a result, it performs particularly well in complex environments. When this technology is applied to incubation eggs—a category of eggs with special requirements for preservation and detection—the mechanical stimulation involved in acoustic signal generation needs to be adjusted. Since incubation eggs contain developing embryos that are sensitive to external mechanical forces, the acoustic detection process must adopt lower-energy excitation methods (e.g., reducing impact intensity or optimizing vibration frequency). This modification ensures that the technology maintains its crack-detection accuracy while avoiding damage to the embryo and compromising its viability, making acoustic detection applicable to both ordinary edible eggs and specialized incubation eggs.

Compared to image processing, acoustic detection offers higher sensitivity for identifying microcracks, providing more reliable data for detecting small cracks. For example, Kertész et al. [77] developed a novel acoustic spectroscopy method that detects eggshell cracks by analyzing the frequency spectrum of sound waves. The method achieved an error rate as low as 2.1%, demonstrating the significant potential of this technology for industrial applications [77]. Lashgari and Mohammadigol [76] proposed an acoustic signal-based detection method that analyzes the rolling egg and impact response signals. By integrating Artificial Neural Networks (ANN), the method efficiently classifies cracked eggs, achieving an accuracy rate of 92.3% [76]. Wang et al. [65] utilized both frequency-domain and time-domain features of acoustic signals, combined with neural network analysis, to achieve high-precision detection of cracks in poultry eggs [65]. Lai et al. [63] proposed a crack detection method for duck eggs based on acoustic emission signals. By measuring the sound wave frequency and integrating logistic regression analysis, they achieved efficient prediction of duck egg cracks, with an accuracy rate of 87.6% [63]. Deng et al. [78] employed acoustic pulse response combined with Support Vector Machine (SVM) to classify cracks in poultry eggs. They first applied a slight impact to the eggshell and recorded the resulting acoustic signals, then extracted frequency-domain features such as peak frequency and resonance frequency. Finally, they used SVM for classification. Experimental results showed that the method effectively identified cracked eggs, achieving a detection accuracy of 98.18% and a false rejection rate of less than 2.11% [78]. Sun et al. [79] proposed a crack detection method based on acoustic resonance. By applying multiple impacts to different positions of the eggshell and collecting the response signals, they used correlation analysis and Multivariate Analysis of Variance (MANOVA) to process the data, ultimately achieving a crack detection rate of 95.5%. This method effectively distinguishes intact eggs from cracked eggs and demonstrates strong resistance to interference [79]. Yumurtacı et al. [80] proposed a crack detection method based on acoustic signals. By applying mechanical impacts to the eggshell and recording the resulting acoustic response, they used threshold clipping and statistical feature extraction methods, followed by classification of the acoustic signals using common algorithms such as Support Vector Machine (SVM) and Artificial Neural Network (ANN). Experimental results showed that the method achieved an accuracy of 100% in detecting cracked eggs and was capable of real-time eggshell crack detection [80].

Although acoustic detection methods offer high sensitivity and accuracy, they still face challenges in practical applications, such as interference from noise and variations in eggshell thickness, especially in noisy production line environments. To improve accuracy, researchers have gradually combined multi-sensor technology with acoustic detection, using multi-channel signals and data fusion techniques to enhance the robustness of the system.

### 3.4. Multi-Sensor Fusion for Crack Detection

With technological advancements, researchers have begun to combine multiple detection technologies to create integrated detection systems [90]. For example, multimodal detection methods that combine acoustic signals, image processing, and electrical characteristics from various sensor technologies can not only improve the accuracy of crack detection but also effectively overcome the limitations of using a single method [91].

Lin et al. [81] proposed a crack detection method based on multiple vibration sensors. They installed several vibration sensors at different positions on the eggshell and analyzed the signals obtained from each sensor, along with their correlations, to extract crack features and perform classification. Experimental results showed that the method achieved a detection accuracy of 93.75% for cracks and effectively enhanced the system’s resistance to interference [81]. Pan et al. [83] proposed a method for eggshell crack detection using both computer vision and acoustic response systems, combined with back-propagation artificial neural networks (BPANN). The method utilizes features from both systems, achieving a detection accuracy of 98% when the information from the computer vision system (CVS) and acoustic response system (ARS) were fused. The research demonstrates the effectiveness of these integrated technologies for crack detection in eggs [83].

The multi-sensor fusion method, by combining data from different sensors, enables more comprehensive information collection, thereby improving the overall performance of crack detection. Future research is likely to focus on optimizing sensor configurations, enhancing the accuracy of data fusion, and designing more efficient classification algorithms to further improve the performance of detection systems [82].

### 3.5. Electrical Properties for Crack Detection

Electrical property detection methods identify cracks by monitoring changes in the electrical parameters on the eggshell surface. When a voltage is applied to the eggshell, cracks alter the conductive properties of the current. Therefore, by analyzing changes in the current signal, cracks can be effectively detected [92]. This method offers high sensitivity, capable of detecting microcracks, and is less dependent on environmental conditions.

Shi et al. [66] proposed a microcrack detection method based on electrical properties, which combines wavelet scattering transform and Convolutional Neural Networks (CNNs). By extracting high-frequency features from the electrical signals, this method successfully identifies microcracks smaller than 3 μm. Experimental results show that at a voltage of 1000 V, the method achieved an accuracy of 99.44%, maintaining high detection precision even at low signal-to-noise ratios. Additionally, the method significantly reduces the potential harm of high voltage to incubating eggs, meeting industrial application requirements [66]. Joe et al. [84] developed a crack detection device based on the electrostatic discharge phenomenon. By detecting the electrostatic discharge at the crack location under a high-voltage electric field, they successfully achieved efficient detection of cracked eggs. This method leverages the non-uniformity of the electric field; when a crack is present on the eggshell, the conductivity of the cracked area differs from that of the rest of the eggshell, resulting in an electrostatic discharge at the crack, thereby enabling accurate crack detection [84].

The advantage of electrical detection methods lies in their ability to detect microcracks in real-time, and they are not sensitive to ambient lighting or background noise. However, since applying voltage is necessary for testing, researchers are exploring ways to reduce the voltage in order to minimize the potential impact on incubating eggs.

### 3.6. Challenges and Limitations in Industrial Implementation

Despite the remarkable progress in nondestructive crack detection methods, several technological and practical challenges still hinder their widespread industrial deployment. First, many advanced detection techniques—such as terahertz imaging, high-resolution acoustic systems, or deep learning-based classifiers—require complex hardware setups and substantial computational resources, limiting their feasibility for high-speed production lines. Second, variations in eggshell color, texture, and shape across breeds introduce signal variability and reduce detection robustness, especially for microcracks under inconsistent lighting or acoustic conditions. Third, real-time implementation often demands a balance between speed and accuracy, where overly complex models may compromise throughput. Furthermore, high-sensitivity methods such as capacitive or discharge-based systems must address safety concerns for incubating eggs and ensure compliance with food safety standards. Lastly, a lack of standardized datasets and evaluation benchmarks across studies limits cross-platform comparability and hinders technology transfer to industry. Addressing these issues will require not only technological innovation, but also collaboration between researchers, equipment manufacturers, and poultry producers.

In addition to laboratory research, several commercially deployed systems have already been widely adopted for eggshell quality inspection in industrial production lines. For example, MOBA (Netherlands), a leading global supplier of egg grading and packing systems, has developed integrated inspection modules capable of detecting eggshell cracks using advanced acoustic and machine vision technologies. Their equipment, such as the MOBA Omnia series, can process over 200,000 eggs per hour with a crack detection accuracy exceeding 95%, making it highly suitable for high-throughput environments. Similarly, NABEL (Japan) provides industrial egg grading systems equipped with optical and acoustic sensors for real-time crack detection and surface defect classification. The NABEL ESL series, for instance, is widely used in Asia and has demonstrated robust performance under diverse environmental conditions. Notably, many laboratory-based techniques discussed in this review, including acoustic resonance and high-resolution machine vision, are inspired by or integrated into these commercial systems. Including insights from such industrial applications highlights the practical value of nondestructive testing technologies and bridges the gap between experimental studies and real-world deployment.

## 4. Poultry Egg Thickness Detection

Eggshell thickness plays a critical role in determining the strength, protective function, and hatching quality of poultry eggs. Traditional thickness measurement methods often require the destruction of the eggshell, such as using micrometer screw gauges, compression tests, and laser interferometry. While these techniques provide highly accurate data, their destructive nature limits their applicability in real-world production settings.

It is important to note that most of the reviewed techniques assess the thickness of the mineralized calcareous layer of the eggshell. The inner shell membrane is typically not included in these measurements due to its soft structure and minimal influence on acoustic, optical, or electromagnetic signal responses.

**Figure 2:**
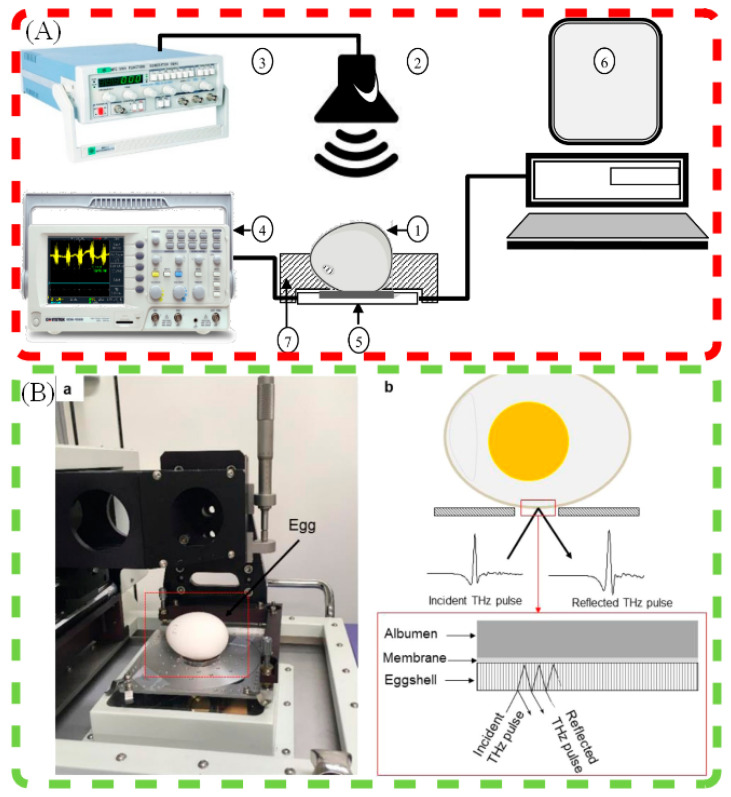
Detection principles of common nondestructive methods for measuring eggshell thickness. (**A**) Schematic diagram of the acoustic resonance-based measurement system for evaluating eggshell strength and thickness. The system consists of a loudspeaker, function generator, digital oscilloscope, piezoelectric sensor, computer, and egg holder. (1) Tested egg, (2) loudspeaker, (3) function generator, (4) digital oscilloscope, (5) piezoelectric sensor, (6) computer, (7) holding sponge [93]. (**B**) Signal (reflected THz pulses) acquisition of intact egg using THz-TDS. (**a**) Actual figure during signal acquisition using THz-TDS. Terahertz waves are electromagnetic waves with picosecond periodic vibrations. Femtosecond lasers were used in the THz-TDS to accurately measure the picosecond pulses. (**b**) Schematic diagram of terahertz reflectance phenomena of intact eggshell. The signal was taken in horizontal position of egg to avoid air sac at upper end of egg and to minimize the degree of curvature of eggshell [94].

As a result, researchers have increasingly turned to nondestructive techniques, which not only improve measurement efficiency but also preserve the integrity of the eggshell for subsequent processing. A comprehensive comparison of nondestructive eggshell thickness detection techniques is provided in Table 3, highlighting their respective principles, parameters, and accuracy levels. To provide a systematic understanding of the measurement strategies, the detection principles of several commonly used nondestructive methods for eggshell thickness evaluation are illustrated in Figure 2.

**Table 3:** Comparative summary of eggshell thickness detection techniques and performance metrics.

Type	Technique	Parameters	Model	Accuracy	Explanation	Ref.
Acoustic detection	Non-contact acoustic resonance excitation using mechanical vibration	30 eggs were used in the experiment, Mechanical tap excitation, microphone capture, calibration with compression tests	Linear regression of resonance vs. strength/thickness	Strength: r = 0.97, Thickness: r = 0.91	Uses mechanical vibration to induce resonance and correlates frequency with strength and thickness.	[93]
Ultrasound detection	High-frequency ultrasound wave reflection technique	180 eggs (Bovance breed, freshly produced, randomly sampled), Ultrasound transducer at equator, compared with micrometer readings	Regression vs. control (dial gauge)	Error 7.1%	Measures thickness using ultrasound reflection and compares with traditional micrometers.	[95]
	Ultrasonic scanning at five angular positions (USG0, USG45, USG90, USG135, USG180)	6939 eggs total 4525 Rhode Island White (RIW) 2414 Rhode Island Red (RIR), Commercial USG device, repeated measurements, compared with electronic micrometer	Heritability estimation, multiple-trait model	Repeatability >0.90, Heritability up to 0.23	Scans eggshell thickness at multiple positions to ensure high reliability.	[96]
Terahertz spectroscopy	Terahertz (THz) reflectance spectroscopy in the frequency domain	THz wave pulse (~0.2–1.2 THz), analyzed with linear regression using 1/Δf	Linear regression (1/Δf)	R^2^ = 0.93, RMSEP = 0.009	Uses THz waves to measure thickness by analyzing the reflected frequency spectrum.	[94]
	Time-domain THz spectroscopy using fiber-coupled source (0–4 THz)	Twelve egg samples were used, THz time-domain signal, verified via FESEM imaging	Spectral analysis + FESEM validation	By comparing the results with FESEM experiments, the method has been demonstrated to be relatively accurate and reliable.	Analyzes the time-domain THz signal to derive thickness and dielectric properties.	[97]
Spectroscopy	Transmission VIS/NIR spectroscopy with preprocessing (MSC, derivatives)	The sample size was 70 eggs., VIS-NIR spectra (300–1100 nm) at equator, PLS regression	PLS (R^2^ = 0.84)	RMSE = 0.01 mm	Uses VIS/NIR spectroscopy to measure thickness with preprocessing techniques.	[98]
	Near-infrared diffuse reflectance spectroscopy	a total of 88 pink-shelled eggs, Spectral data at three egg positions, PLS modeling with derivatives	PLS with preprocessed spectral data	Equator R = 0.69, RMSE ≈ 0.02 mm	Measures regional thickness using near-infrared spectroscopy with derivative preprocessing.	[99]

### 4.1. Acoustic Resonance Method

Attar and Fathi et al. [93] proposed the acoustic resonance method, which evaluates eggshell thickness by analyzing changes in the resonance frequency of the shell when subjected to acoustic excitation [93]. This method measures the egg’s vibrational characteristics across different frequencies to determine the dynamic stiffness of the shell. The study found a significant correlation between the resonance frequency and both the thickness and strength of the eggshell. As the eggshell becomes thinner, its frequency response changes—inversely proportional to the shell thickness. The main advantage of this method is that it enables rapid, nondestructive measurement of eggshell thickness while also providing information related to shell strength. By employing this approach, researchers can efficiently screen for high-quality eggshells, supporting quality control and industrial-scale egg production.

In addition, the acoustic resonance method has been further extended to assess eggshell strength, particularly in industrial-scale shell inspection. Excitation signals at varying frequencies can provide more detailed information about both the hardness and thickness of the eggshell, thereby enhancing the accuracy of eggshell quality control.

### 4.2. Ultrasonic Measurement

The ultrasonic method [100] estimates eggshell thickness by analyzing the propagation speed and attenuation characteristics of ultrasonic waves within the shell [95]. Compared to traditional destructive measurement methods, the ultrasonic technique not only enables precise thickness measurement but also allows for real-time assessment of eggshell uniformity and quality. The principle of this method is that the speed of ultrasonic wave propagation through the eggshell is related to its density and elasticity. By analyzing the reflected ultrasonic signals, the thickness of the eggshell can be calculated. Compared to conventional methods, the ultrasonic approach offers lower operational complexity, higher measurement accuracy, and the advantage of being non-destructive. As a result, it is widely applied in modern poultry egg production, particularly in quality screening and grading processes.

Sirous Amini et al. [95] proposed a non-destructive ultrasonic method to measure the thickness and strength of eggshells. They compared ultrasound measurements with other methods such as photometry, micrometer, and dial gauge. The results indicated that ultrasound provided the most accurate measurements, with an error of only 7.1%, compared to 15.7% for the photometric method. Furthermore, they established a regression equation that showed a positive correlation between eggshell strength and thickness, confirming the efficacy of ultrasound for quality grading in the egg industry [95]. Kibala et al. [96] proposed a methodology using ultrasonic technology (USG) to measure eggshell thickness for layer selection in poultry. They measured eggshell thickness at five different latitudes (USG0, USG45, USG90, USG135, and USG180) using ultrasonic devices and found a high genetic correlation between eggshell thickness and egg strength, with a repeatability above 0.90 for measurements at USG45. The study demonstrated that USG45 was the most reliable measurement for balancing hatchability and shell strength, with heritability of shell thickness ranging from 0.09 to 0.23 for different latitudes. The results support the use of USG45 as a selection criterion in breeding programs for improving eggshell quality [96].

### 4.3. Terahertz Spectroscopy Technology

Terahertz (THz) waves [101] are used to estimate eggshell thickness by analyzing the reflected signals at the internal and external interfaces of the shell. Unlike traditional visible and infrared light, terahertz waves possess strong penetration capabilities, allowing them to pass through the eggshell surface and probe different layers of the shell. Specifically, the THz Time-Domain Spectroscopy (THz-TDS) technique establishes a mathematical model between eggshell thickness and the reflected signal in the frequency domain by analyzing the waveform reflected from the eggshell and inner membrane. Studies have shown that THz-TDS can provide high-resolution thickness estimations with an accuracy of up to 10 μm, and the technique has demonstrated wide applicability across different types of eggshells. In addition, THz-TDS can simultaneously provide optical and dielectric parameter data, which contributes to a deeper analysis of the eggshell’s physical properties and quality [102]. Therefore, terahertz spectroscopy has emerged as a promising nondestructive technique for poultry eggshell thickness detection. When applied to incubation eggs, THz systems must ensure thermal safety and non-interference with embryonic development.

Khaliduzzaman et al. [94] proposed a non-destructive method to measure eggshell thickness using terahertz (THz) waves. By analyzing the reflected THz pulse from both the air-eggshell interface and the inner shell-membrane interface, the researchers developed a linear regression-based model to estimate eggshell thickness. The model achieved a coefficient of determination (R^2^) of 0.93, with a root mean square error of prediction (RMSEP) of 0.009, and provided resolution down to less than 10 μm. This method demonstrates excellent prediction performance and can be applied to industrial egg grading [94]. Iram et al. [97] proposed a non-destructive method for measuring eggshell thickness, optical properties, and dielectric parameters using Time-domain Terahertz (THz) Spectroscopy. They compared the refractive indices and absorption coefficients of table and fertile eggs and found significant differences between the two types of eggs. The study also included measurements of the real and imaginary parts of dielectric constants, which showed that fertile eggs had a more consistent response, while non-fertile eggs demonstrated a steep drop in values. The method proved highly effective in characterizing eggshells and holds potential for use in industrial egg grading [97].

### 4.4. Visible and Near-Infrared Spectroscopy (VIS/NIR) Technology

Dong Xiaoguang et al. [98] proposed a nondestructive eggshell thickness detection method based on visible/near-infrared (VIS/NIR) transmission spectroscopy. They applied multiple spectral preprocessing techniques, including Savitzky–Golay smoothing, multiplicative scatter correction (MSC), and standard normal variate (SNV), and built partial least squares regression (PLSR) models for thickness prediction. Among them, the PLSR model with MSC preprocessing performed best, achieving correlation coefficients of 0.86 and 0.84 in the calibration and prediction sets, respectively, with a root mean square error of 0.01. The study demonstrated the effectiveness of VIS/NIR spectroscopy combined with PLSR for nondestructive eggshell thickness assessment [98]. Han Yawen et al. [99] proposed a nondestructive method for detecting eggshell thickness in different egg regions using near-infrared (NIR) diffuse reflectance spectroscopy. They applied original spectral data, first-order, and second-order derivatives as preprocessing methods and constructed partial least squares regression (PLSR) models to predict shell thickness at the large end, equator, and small end of the eggs. Results showed that the model using raw spectral data for the equator region achieved the most stable performance, with a correlation coefficient of 0.6913 and root mean square errors of 2.0632 and 2.0194 (0.01 mm units) for the calibration and prediction sets, respectively. Although the model demonstrated feasibility for nondestructive detection, its quantitative prediction accuracy was relatively low, indicating the need for further improvement in model precision and robustness against overfitting [99].

### 4.5. Comparative Summary of Thickness Detection Methods

Each nondestructive technique for eggshell thickness detection offers unique advantages and limitations. Acoustic resonance methods are cost-effective, simple to implement, and suitable for industrial integration, but may suffer from signal variability due to shell geometry and background noise. Ultrasonic detection provides high accuracy and real-time monitoring capabilities, yet requires contact-based transducers and coupling agents, which may reduce throughput. Terahertz spectroscopy offers exceptional resolution and can capture both thickness and dielectric properties, but the system cost and complexity remain barriers for commercial adoption. VIS/NIR spectroscopy enables rapid, non-contact assessment with relatively simple setups, but its prediction accuracy is lower and highly sensitive to shell surface properties. A comprehensive understanding of these trade-offs is essential for selecting appropriate technologies in different production contexts.

## 5. Poultry Egg Strength Detection

Eggshell strength is a critical factor in determining whether an egg can withstand physical impacts during transportation, handling, and storage. Shell strength not only affects the breakage rate but is also closely linked to quality in processes such as incubation and storage. Traditional methods for measuring eggshell strength are mostly destructive; while accurate, they are unsuitable for large-scale, high-efficiency production lines. As a result, nondestructive detection technologies are playing an increasingly important role in modern eggshell quality control. The following are several major nondestructive techniques currently in use. The relative performance and characteristics of these strength detection methods are compared in Table 4.

### 5.1. Ultrasonic Method

Ultrasonic techniques assess the mechanical properties of eggshells by measuring wave velocity and attenuation characteristics. Studies have shown that compared to traditional static compression testing, the ultrasonic method yields smaller measurement errors and can provide rapid, real-time data. It not only avoids damaging the eggshell during the measurement process but also adapts well to high-efficiency production environments. Additionally, ultrasonic wave speed is closely correlated with eggshell thickness and strength, making it possible to infer shell quality from ultrasonic signals—thus offering valuable support for quality screening in large-scale production.

Amini et al. [95] investigated an ultrasonic-based method for measuring the strength of poultry eggshells. The results indicated that ultrasonic technology can nondestructively evaluate both the strength and thickness of the eggshell [95].

The advantages of ultrasonic methods lie in their high efficiency and accuracy, making them well-suited for egg quality grading, especially in automated production lines. This technique is characterized by low cost, fast measurement speed, and ease of operation, making it one of the leading nondestructive measurement technologies currently in use.

### 5.2. Acoustic Resonance Method

**Figure 3:**
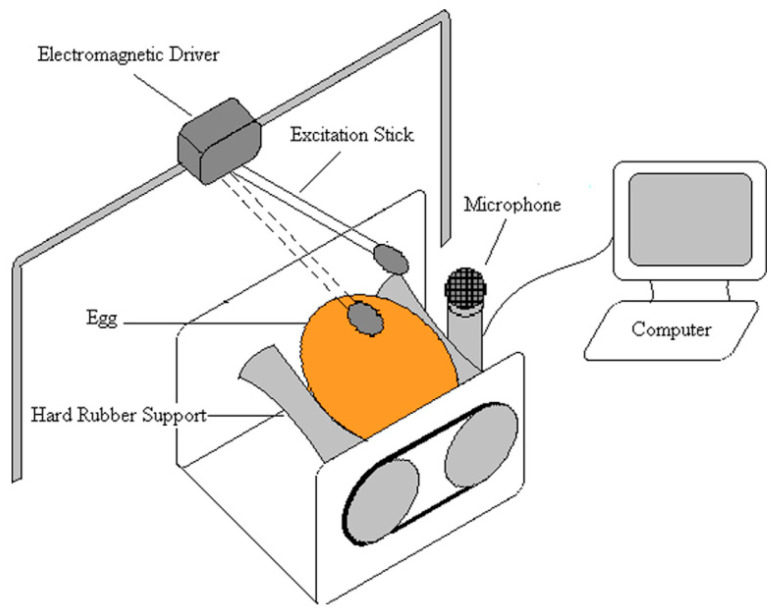
Schematic diagram of the nondestructive eggshell stiffness measurement system based on acoustic resonance. The system includes a mechanical excitation device, a microphone, signal amplifiers, and a data acquisition module for analyzing eggshell vibration responses [103].

Attar and Fathi [93] proposed a nondestructive method based on acoustic resonance, which evaluates eggshell strength by measuring its resonance frequency. In this approach, the eggshell is excited to induce resonant vibrations, and its natural frequency is measured. The resonance frequency is then used to estimate the shell’s strength. Studies have shown a strong correlation between the eggshell’s resonance frequency and its mechanical strength. Compared to traditional static strength testing methods, this technique offers faster and nondestructive measurement of eggshell strength [93]. Hao Lin et al. [103] proposed a non-destructive method for measuring eggshell stiffness using an acoustic resonance system combined with partial least squares (PLS) regression models (Figure 3). They compared several frequency selection algorithms, including conventional PLS, synergy interval PLS (si-PLS), genetic algorithm PLS (GA-PLS), and GA-siPLS. Among these, the GA-PLS model achieved the best performance with a prediction set correlation coefficient of 0.771 and a root mean square error of prediction (RMSEP) of 3.6. The study demonstrated that acoustic resonance combined with optimized multivariate models enables compact, robust, and efficient measurement of eggshell stiffness for potential use in real-time industrial applications [103].

The main advantages of the acoustic resonance method are its simplicity and ability to provide strength information without damaging the eggshell. By employing this technique, production lines can perform real-time quality inspection to prevent high breakage rates. It does not require complex equipment and can evaluate eggshell strength using simple frequency response signals. Additionally, this method offers high precision and stability, making it a valuable tool for quality control during the production process.

### 5.3. Hertzian Contact Theory Method

De Ketelaere et al. [104] proposed a non-destructive eggshell strength assessment method based on Hertz contact theory, utilizing a small steel impactor and a microphone to record the bouncing sound after impact. Key parameters such as contact time and impact speed were extracted from the microphone signal to calculate the Hertz stiffness. The study found a strong correlation (r = 0.93) between the Hertz stiffness and the static stiffness measured by quasi-static compression, and a similarly high correlation with shell thickness (r = 0.88). This low-cost, fast, and accurate method proved suitable for real-time, in-line applications, offering potential for individual egg grading and early detection of poultry health issues in production environments [104].

The advantage of the Hertzian contact theory method lies in its ability to quickly and accurately assess eggshell strength using simple equipment. This method is particularly suitable for efficient eggshell quality screening and can be integrated into automated production lines for real-time monitoring. In this way, producers can more effectively identify eggs with higher shell strength, thereby reducing the risk of damage.

### 5.4. Combination of NIR Spectroscopy and Artificial Intelligence (AI)

Ahmed et al. [105] proposed a novel nondestructive method for predicting eggshell strength by integrating Near-Infrared (NIR) spectroscopy with interpretable artificial intelligence (AI) techniques. The approach involves scanning eggshells using NIR spectroscopy and developing predictive models through machine learning algorithms such as Partial Least Squares Regression (PLSR) and Random Forest (RF). To enhance interpretability, the study employed Shapley Additive Explanations (SHAP), which quantify the contribution of individual spectral features to the model’s predictions. This method demonstrated strong predictive performance, achieving an R^2^ of 0.83 and a root mean square error (RMSE) of 1.49 N [105].

The key advantage of combining NIR spectroscopy with AI lies in its ability to deliver nondestructive, efficient, and interpretable strength assessments [106]. It enables real-time analysis and helps producers identify critical factors affecting eggshell strength, supporting targeted adjustments during production. Due to its high accuracy and speed, the method shows great potential for large-scale industrial implementation.

### 5.5. Practical Limitations and Industrial Challenges

While various nondestructive techniques for eggshell strength detection have demonstrated promising accuracy in laboratory settings, several practical challenges limit their industrial-scale implementation. First, contact-based methods such as ultrasonic or Hertzian impact testing may require precise alignment and coupling media, reducing their applicability in high-speed sorting lines. Second, environmental variability—such as changes in temperature, humidity, and vibration on production lines—can affect the consistency of acoustic and ultrasonic measurements. Third, techniques like spectroscopy combined with machine learning require extensive calibration and large training datasets to maintain generalization across different egg types, shell colors, and production conditions. Furthermore, balancing real-time performance with computational complexity remains a challenge, especially when deep learning models are involved. Lastly, the lack of standardized validation protocols for strength detection makes it difficult to benchmark and deploy these systems uniformly across production facilities. Addressing these issues will be critical for transitioning from experimental validation to full industrial adoption. Some strength detection methods may be more suited to table eggs than fertilized incubation eggs, where embryonic safety is critical.

## 6. Detection of Eggshell Color and Cleanliness

The external quality of poultry eggs—particularly the color and cleanliness of the eggshell—is a key factor affecting market value, consumer acceptance, and overall food safety. As consumer concern over food quality and safety continues to grow, traditional manual inspection methods are increasingly unable to meet the efficiency and precision demands of the modern food processing industry. Consequently, automated detection systems based on computer vision technology have been widely adopted. These systems have demonstrated significant potential in areas such as color assessment, stain detection, and crack identification in poultry eggs. A comparison of representative techniques for eggshell color and cleanliness detection, including their accuracy is provided in Table 5.

### 6.1. Color Detection and Classification

Eggshell color is an important factor influencing consumer choice. It is closely related not only to the breed of the hen, rearing environment, and nutritional intake, but also to certain aspects of egg quality, shell strength, and storability.

Vasileva et al. [107] developed a machine vision-based method for evaluating the external quality of chicken eggs. This method integrates candling and image acquisition to automatically assess parameters such as geometric dimensions, shape index, and mottling grade. Using a dataset of 400 eggs, the authors designed an image processing workflow and compared the automated results with manual assessments. The shape index demonstrated a strong correlation between both methods (r = 0.93), and the F-measure for mottling classification reached 0.921. The system effectively detected shape anomalies and microcracks, showing strong potential for automated egg grading [107].

Gorbunova et al. [108] introduced a contactless quality control system based on machine vision, combining both reflection and transmission optical modes. Their electro-optical prototype was equipped with software capable of analyzing eggshell color, geometric dimensions, and surface defects such as cracks, pores, and marbling. Validated using spectrophotometric analysis and tested on over 300 eggs from various chicken breeds, the system demonstrated high consistency and reliability, supporting its application in rapid, nondestructive egg quality assessment for breeding and industrial sorting [108].

Drabik et al. [109] examined the relationship between eggshell color, mineral composition, and overall egg quality. Eggs from three breeds—Araucana (green), Marans (brown), and Leghorn (white)—were analyzed using the CIE Lab* color space and atomic absorption spectrometry (AAS) to quantify minerals such as calcium (Ca), magnesium (Mg), and zinc (Zn). The results showed that eggshell color significantly affected shell strength and thickness, with dark brown eggs having the highest shell strength and calcium content (*p* < 0.001). In contrast, white-shelled eggs exhibited better performance in egg weight and albumen content. Furthermore, strong correlations were found between color coordinates (e.g., L*, a*) and mineral levels, indicating that eggshell color reflects both visual appearance and underlying structural properties [109].

### 6.2. Cleanliness Detection

Eggshell cleanliness is another critical quality indicator. Dirty eggshells not only affect visual appeal and reduce market value, but also pose potential food safety risks, especially when the shell is contaminated with bacteria or viruses. Traditional cleanliness inspection methods typically rely on manual visual inspection; however, due to human eye fatigue and high susceptibility to environmental factors, such manual approaches are often inefficient and prone to error.

Beyaz et al. [110] developed a dirt detection system based on image analysis technology for the automated identification of stains on eggshell surfaces. The system utilizes image acquisition and processing techniques to detect contaminants such as feces, uric acid, egg yolk, and blood. The image analysis software, developed on the LabVIEW platform, can accurately classify eggs based on the color and shape of the stains, enabling precise identification of eggs with low cleanliness. The study showed that the system achieved an identification accuracy of up to 99.8% for various types of stains and was capable of rapidly screening dirty eggshells on production lines, thereby significantly improving quality control efficiency [110].

Eggshell mottling—particularly stripe-like patterns—is closely associated with the presence of hairline cracks. These mottling areas are typically more translucent and structurally weaker, making them prone to microfracture development during production or pasteurization. Wong et al. [112] employed Convolutional Neural Networks (CNNs) to identify irregular stains on eggshells and proposed a deep learning-based method for dirt detection. The approach utilizes a pre-trained CNN model to classify stain patterns on the eggshell and compares the results with human evaluations, achieving a classification accuracy of 91.8%. This CNN-based method effectively handles complex stain patterns and offers good interpretability, providing strong technical support for the automated detection of eggshell cleanliness [112].

Wang et al. [111] proposed a machine vision-based method for detecting dark spots on eggshells. The method applies the K-means clustering algorithm to segment the eggshell image and uses an unsharp masking technique to enhance dark spot features, enabling automatic identification and quantitative assessment. By rapidly processing images, the system allows for real-time detection of dark spots on the eggshell surface. It evaluates cleanliness and quality by calculating the area ratio of the spots. Compared to manual annotation, this method improved efficiency by 960 times and is capable of processing 3600 eggs per hour, demonstrating significant advantages [111].

**Figure 4:**
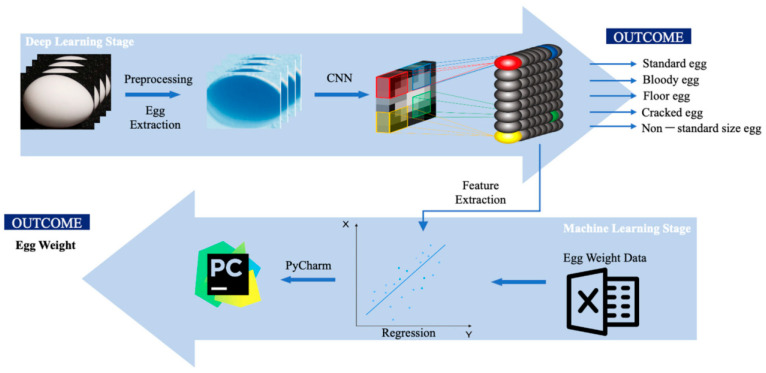
Workflow of egg quality classification using a computer vision-based system. The process integrates deep learning for egg classification and random forest regression for weight prediction based on extracted image features [113].

Yang et al. [113] proposed an automatic egg grading and defect detection system based on deep learning, integrating Real-Time Multi-Task Detection (RTMDet) with the Random Forest (RF) algorithm. The system can simultaneously detect external defects, grade eggs, and predict their weight. It accurately identifies surface defects such as cracks, blood spots, and stains, significantly improving the efficiency of egg sorting. As shown in Figure 4, the proposed computer vision-based system follows a streamlined workflow that combines deep learning for egg classification and regression models for weight prediction [113].

### 6.3. Limitations of Current Technologies

Although computer vision and deep learning-based technologies have greatly improved the accuracy and automation of eggshell color and cleanliness detection, several limitations remain. First, most vision-based methods are highly sensitive to lighting conditions and background interference. Inconsistent illumination, shadows, or reflections on the eggshell surface can lead to false detections or reduced classification accuracy. Second, surface stains and natural mottling patterns are often difficult to distinguish, especially in the presence of hairline cracks or shell texture variations, which may mislead defect recognition models. Third, deep learning approaches typically require large, well-annotated datasets for training and may suffer from performance degradation when applied to eggs from different breeds or production environments. Additionally, real-time processing demands high computational power, which may hinder deployment on cost-sensitive production lines. Finally, the lack of standardized criteria for evaluating shell cleanliness and color grading adds subjectivity to the system outputs, limiting comparability across different facilities. Overcoming these limitations requires improvements in lighting control, dataset generalization, and interpretability of AI-based systems.

## 7. Detection of Other Indicators

In poultry egg quality assessment, beyond the commonly evaluated external indicators such as shell cracks, thickness, strength, color, and cleanliness, other physical properties—including egg volume, surface area, shape, and texture—also significantly impact market value and functional performance. With the continuous advancement of technology, particularly the application of computer vision, ultrasound, and deep learning, nondestructive methods have made notable progress in measuring these additional indicators. The following provides a detailed review of recent research developments in the detection of these important quality attributes of poultry eggs. As summarized in Table 6, these emerging nondestructive techniques provide valuable complementary information beyond conventional structural indicators.

### 7.1. Nondestructive Measurement of Egg Volume and Surface Area

Egg volume and surface area are important parameters for evaluating overall egg quality, particularly in relation to shape, structural strength, and transport stability. Traditionally, the measurement of egg volume and surface area has relied on destructive testing methods, which are unsuitable for real-time quality control in large-scale production.

**Figure 5:**
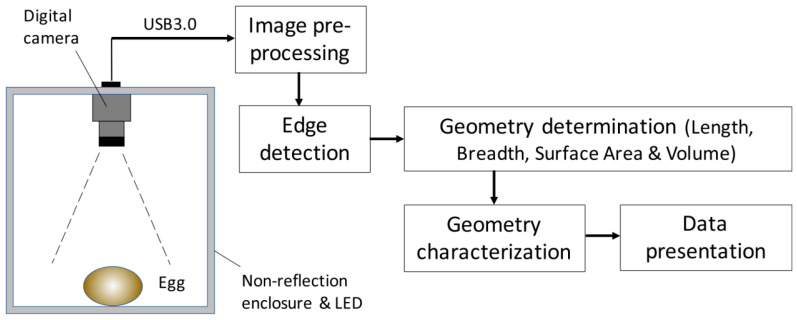
Block diagram of the 2-D imaging system for measuring egg geometrical parameters. The system integrates a CMOS camera, LED-based non-reflective lighting, and a computer-based image processing module for extracting key morphological features such as length, area, and breadth [114].

To address this limitation, Narushin et al. [114] as illustrated in Figure 5 proposed a nondestructive method based on two-dimensional image-based geometric transformations to estimate egg volume and surface area (Figure 5). By digitally capturing the geometric shape of the eggshell and applying model transformations using egg shape models (such as ellipsoids or ovoidal models), this method enables rapid and accurate measurement of egg volume and surface area without damaging the shell. The results demonstrated that this approach can generate precise models of eggshell geometry and has been widely applied in egg classification, packaging, and quality monitoring [114].

In addition, an image-processing-based technique has been developed that integrates 3D scanning technology with machine learning algorithms [119] to enable real-time monitoring of egg volume and surface area. This method employs high-resolution image acquisition and data analysis to provide accurate, contactless volume measurements, thereby minimizing the risk of eggshell damage.

### 7.2. Egg Shape Index and Mechanical Properties

The Shape Index (SI) [120], defined as the ratio of egg width to length, is one of the standard indicators used to evaluate egg morphology. Studies have shown that the SI not only influences the visual appearance and packaging compatibility of eggs but is also closely related to mechanical properties such as shell strength and compressive resistance. Duman et al. [115] found that eggs with a more standard shape (SI between 72 and 76) generally exhibit better resistance to compression and lower breakage rates. In contrast, irregularly shaped eggs—those that are overly round or elongated—are more prone to cracking during transportation and storage [115]. Mota-Grajales et al. [116] proposed an eggshell defect detection method based on laser scanning and image processing. By scanning the eggshell surface with a laser beam and integrating computer vision techniques, the system is capable of detecting and classifying various types of defects, such as dents, cracks, and irregular shapes. The method constructs geometric curves and performs interpolation analysis to accurately identify shell deformations, and utilizes an Artificial Neural Network (ANN) for defect classification. Experimental results demonstrated that this method offers high detection efficiency and provides real-time support for egg quality control [116]. Altuntas et al. [117] proposed a method to assess eggshell mechanical properties based on shape index (SI). Results showed that eggs with higher SI had greater rupture force and stiffness, especially along the X-front axis, supporting its use in strength-based grading [117].

Furthermore, research indicates that SI is also correlated with internal quality traits such as shell thickness, albumen quality, and hatching performance. Eggs with higher SIs typically possess stronger shells, enabling them to better withstand mechanical stress during production and transport, thereby reducing breakage rates. Measuring the shape index provides an effective means of assessing eggshell quality and offers a scientific basis for egg grading and packaging.

### 7.3. Eggshell Texture Features and Individual Identification

**Figure 6:**
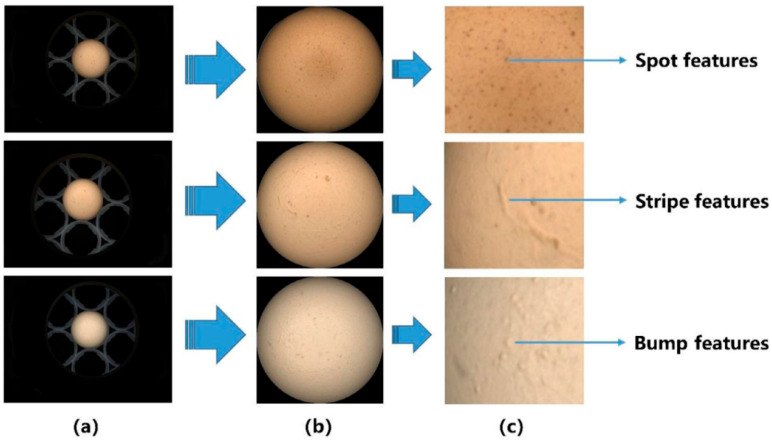
Eggshell image samples and representative texture features after preprocessing. The figure shows the original images (**a**), binarized and segmented eggshell regions (**b**), and the typical surface textures (spots, stripes, and bumps) (**c**) used for biometric identification [118].

In recent years, with the advancement of deep learning technologies, computer vision has been widely applied to the extraction of eggshell texture features and individual egg identification [121]. As shown in Figure 6, the eggshell surface contains rich and diverse natural texture patterns, which are extracted after image preprocessing and used as unique identifiers in biometric models.

Chen et al. [118] proposed a deep learning-based method for identifying individual eggs based on their texture features. This approach employs Convolutional Neural Networks (CNNs) to extract detailed texture information from the eggshell surface, enabling the unique differentiation of each egg. Experimental results showed an identification accuracy of 99.96%, highlighting the method’s strong potential for applications in egg traceability and anti-counterfeiting systems [118].

This method leverages the natural texture patterns on eggshells—such as spots and stripes—to assign a unique identity to each egg. This has important implications for traceability, quality monitoring, and anti-counterfeiting. Particularly in egg production and distribution, using these texture features for individual identification enables precise origin tracking and quality control.

### 7.4. Practical Significance and Limitations

The detection of secondary eggshell indicators such as shape index, surface roughness, pore structure, and texture features plays an increasingly important role in quality classification, traceability, and hatchability prediction. These indicators provide valuable complementary information beyond basic structural parameters, enabling more refined quality control and grading strategies. For example, eggshell pore characteristics are closely linked to gas exchange during incubation, while surface roughness affects disinfection effectiveness and microbial contamination risk. Moreover, eggshell texture analysis can support individual egg identification and traceability systems.

However, the practical implementation of these methods faces several challenges. Many require high-resolution imaging equipment, controlled environmental conditions, or complex algorithms that limit real-time performance and scalability. Techniques such as SEM or 3D laser scanning are typically confined to laboratory use due to cost and speed limitations. Furthermore, the lack of standardized benchmarks and limited integration into current industrial equipment make it difficult to deploy these technologies in commercial production lines. To fully realize their potential, future work should focus on developing simplified, fast, and low-cost alternatives with strong generalization capabilities and compatibility with existing egg sorting systems.

## 8. Challenges and Future Trends

Current nondestructive testing (NDT) methods for eggshell quality face key challenges in sensitivity, noise reduction, and variability. Detecting microcracks under 3 μm requires highly sensitive sensors, yet such weak signals are easily masked by background noise, as noted in capacitive and corona discharge studies. High-speed imaging and spectroscopy also struggle with motion blur and lighting changes on conveyor belts, while surface variations (e.g., speckles, gloss) can lead to false positives in visual inspection.

System cost and complexity pose further barriers. Techniques like Raman spectroscopy and hyperspectral imaging offer high accuracy but are expensive and unsuitable for high-throughput environments. Industrial applications demand real-time results—processing thousands of eggs per hour—yet current systems often require intensive computation. Compact, embedded sensors with onboard AI are being explored to meet this need.

Lack of standard datasets and protocols also limits method comparability. Most studies rely on custom setups, making it hard to evaluate performance across platforms. Future directions include integrating AI and explainable models to improve accuracy and transparency. Shapley-based NIR models, for example, reveal key spectral features for shell assessment. Sensor fusion—combining imaging, acoustic, and electrical data—can provide comprehensive quality fingerprints. Edge computing and hardware-software co-design will also be vital for building scalable, real-time inspection systems.

To move the field forward, several research priorities should be emphasized. First, there is a need for standardized, high-quality public datasets across multiple egg types and detection conditions to enable robust model development and cross-study comparisons. Second, developing lightweight and interpretable AI models that can be embedded on edge devices is critical for real-time, in-line implementation. Third, greater emphasis should be placed on multimodal sensor fusion, enabling systems to compensate for individual modality limitations and improve detection robustness. Additionally, the development of low-cost, scalable hardware solutions—especially for small and medium-sized enterprises—should be prioritized to enhance technology accessibility. Finally, establishing industrial-grade validation protocols and creating adaptable, modular software frameworks will be key to facilitating large-scale deployment and regulatory acceptance.

## 9. Conclusions and Future Directions

Recent advances have significantly improved the accuracy of eggshell NDT methods, with some reaching 96–99%. However, scaling these systems for industrial use requires solutions to noise, variability, and speed constraints. Future platforms must be robust, fast, and interpretable, capable of integrating seamlessly into production lines. Collaboration across disciplines is essential. Public datasets, standardized metrics, and transparent benchmarks will accelerate development and ensure comparability. At the same time, explainable AI and sensor fusion will support regulatory compliance by offering both accuracy and traceability. Ultimately, the future of eggshell NDT lies in compact, integrated systems with onboard intelligence. By aligning research with real-world needs, the industry can achieve automated, reliable, and high-throughput inspection to ensure food safety and quality.

In future research, particular attention should be given to closing the gap between laboratory performance and real-world industrial deployment. For example, portable and low-power sensor modules integrated with real-time AI models can support on-site quality monitoring in egg farms and processing centers. Furthermore, the adoption of explainable machine learning models will help improve user trust and facilitate compliance with food safety regulations. Collaboration between researchers, equipment manufacturers, and poultry industry stakeholders is essential for aligning technological advances with practical requirements. By addressing cost, reliability, and integration challenges, these efforts will accelerate the development of scalable, intelligent, and standardized nondestructive eggshell inspection systems for industrial use.

It is also important to note that table eggs and incubation eggs differ in their quality requirements and constraints. While table eggs primarily focus on cleanliness, color, and mechanical strength to ensure visual appeal and safety, incubation eggs emphasize parameters such as shell porosity, uniformity, and gas exchange efficiency. Consequently, detection techniques must be tailored accordingly. For example, high-voltage electrical methods may be unsuitable for the incubation of eggs due to potential embryonic damage, whereas visual quality and surface defects are more critical for table eggs. Future research should distinguish the application scopes of these technologies based on egg type and processing context.

## Figures and Tables

**Table 1:** Nutritional Information for One Egg (~50 g).

Nutrient	Amount Per Serving	% Daily Value
Calories	~72 kcal	
Total Fat	~5 g	~6%
Cholesterol	186 mg	~62%
Sodium	~62–70 mg	~3%
Total Carbohydrate	~0.4–0.6 g	<1%
Vitamin D	~1 μg (estimated)	~5–7%
Calcium	~28 mg (estimated)	~2–3%
Iron	~1 mg (estimated)	~5–6%
Potassium	~69–70 mg (estimated)	~2%
Protein	~−6.0–6.3 g	~12%

% Daily Value (%DV) is based on a 2000-calorie diet. Note: Nutrient values are obtained from the U.S. Department of Agriculture (USDA) FoodData Central (https://fdc.nal.usda.gov/food-details/748967/nutrients, accessed on 16 August 2025).

**Table 4:** Comparative summary of eggshell strength detection techniques and performance metrics.

Type	Technique	Parameters	Model	Accuracy	Explanation	Ref.
Ultrasonic detection	Non-destructive ultrasonic analysis	180 eggs; Ultrasound transducer; Compared with micrometer, dial gauge, and photometric methods	Linear regression	Consistent with destructive method	Uses ultrasound wave propagation to measure shell thickness and infer strength	[95]
Acoustic detection	Acoustic resonance analysis	110 eggs; Mechanical tapping; Frequency range 1000–8000 Hz; FFT and power spectral analysis	PLS, iPLS, GA-PLS, GA-siPLS	Best model R = 0.771, RMSEP = 3.6	Analyzes frequency response to estimate shell stiffness	[103]
	Impulse excitation acoustic resonance	30 eggs; Hammer tap; Frequencies of shell, albumin, yolk analyzed	Multiple linear regression	Correlation evident	Uses tapping hammer and microphone to collect resonance signals	[93]
Hertzian contact theory	Hertz contact theory-based test	150 eggs; Steel ball (3 g, r = 4.5 mm); 50 kHz sampling	None (direct stiffness estimation)	R = 0.93 (vs. static stiffness)	Uses a steel ball and acoustic signal to estimate stiffness	[104]
Spectroscopy	Near-infrared spectroscopy with machine learning	145 commercial eggs; Bruker TANGO FT-NIR spectrometer; Spectral preprocessing and machine learning	Random Forest (RFE)	R^2^_p_ = 0.83, RMSEP = 1.49 N, RPD = 2.44	Combines NIR spectroscopy with machine learning to predict strength	[105]

**Table 5:** Comparative summary of eggshell color and cleanliness detection techniques and performance metrics.

Detection Index	Type	Technique	Parameters	Model	Accuracy	Explanation	Ref.
Color, Shape, Surface Defects	Image Processing	Image thresholding and candling-based assessment	400 eggs; shape index and mottling	None	Correlation (shape index) 0.93, SD diff 1.05%	Combines image processing with candling for accurate assessment	[107]
		Chromatic and geometric analysis	The sample size was over 300 eggs, Color uniformity, egg dimensions, shape parameters	None	Effective for fine crack and marbling detection	Uses spectral measurements for detailed defect detection	[108]
	Colorimetric Analysis	CIE Lab* analysis and elemental correlation	180 eggs from different breeds; shell traits	Statistical correlation	Breed and color influence mineral content and strength	Analyzes shell color and mineral composition	[109]
Cleanliness detection	Image Processing	LabVIEW-based image analysis	100 clean vs. 100 dirty eggs; feces-based dirt analysis	LabVIEW software	99.8% (painted grade), 98.5% (feces stain)	Detects dirt using dark level image analysis	[110]
		K-means clustering and unsharp masking	initial collection of 416 eggs, yielding 360 valid samples, Real-time inspection; surface segmentation	K-means clustering	High-throughput and accurate detection	Detects dark spots using clustering and image enhancement	[111]
	Deep Learning	Pretrained AlexNet CNN model	A total of 1160 eggshell image patches (695 accepted, 465 rejected) were split into 1000 training/validation and 160 test samples, Translucent mottling images; transfer learning	CNN (AlexNet)	91.8% similarity to human graders	Classifies mottling severity using a pretrained model	[112]
		Two-stage AI model (RTMDet + Random Forest)	A dataset of 2100 egg images was created and randomly split into training (80%) and testing (20%) sets, Imaging and weighing system; detects various defects	RTMDet + RF	94.8% classification, 96.0% weight prediction R^2^	Uses deep learning for classification and weight prediction	[113]

**Table 6:** Comparative summary of eggshell other indicators detection techniques and performance metrics.

Detection Index	Technology Type	Technique	Parameters	Model	Accuracy	Explanation	Ref.
Egg Volume and Surface Area	Image Processing	2D Imaging and Geometric Transformation	Digital imaging; fitted with geometric models	Geometric transformation	High correlation with physical measures	Uses 2D imaging to calculate volume and surface area through geometric models	[114]
Egg shape index and mechanical properties	Correlation Study	Shape Index Classification	1563 eggs; digital caliper, micrometer, colorimeter, Haugh unit	Statistical correlation and ANOVA	Significant relations with albumen and yolk indices	Analyzes correlations between shape index and internal quality metrics	[115]
	Image Processing	Laser Line Scanning and Spline Analysis	200 egg images; spline-based metric extraction	ANN	97.5% classification accuracy	Detects surface defects using laser scanning and spline curve analysis	[116]
	Compression Testing	Compression Test on Axes	270 Lohmann eggs; shape index classification	Compression model	Higher SI = higher rupture force	Tests mechanical strength under compression on different axes	[117]
Texture features	Image Processing	CNN-Based Texture Recognition	770 eggs; 7700 images; blunt-end captured	ResNeXt-50	99.96% correct recognition	Uses CNN to recognize individual eggs based on texture features	[118]
